# Role of co-repressor genomic landscapes in shaping the Notch response

**DOI:** 10.1371/journal.pgen.1007096

**Published:** 2017-11-20

**Authors:** Stephen K. K. Chan, Gustavo Cerda-Moya, Robert Stojnic, Kat Millen, Bettina Fischer, Silvie Fexova, Lenka Skalska, Maria Gomez-Lamarca, Zoe Pillidge, Steven Russell, Sarah J. Bray

**Affiliations:** 1 Department of Physiology Development and Neuroscience, University of Cambridge, Cambridge, United Kingdom; 2 Cambridge Systems Biology Centre, University of Cambridge, Cambridge, United Kingdom; 3 Department of Genetics, University of Cambridge, Cambridge, United Kingdom; Stanford University School of Medicine, UNITED STATES

## Abstract

Repressors are frequently deployed to limit the transcriptional response to signalling pathways. For example, several co-repressors interact directly with the DNA-binding protein CSL and are proposed to keep target genes silenced in the absence of Notch activity. However, the scope of their contributions remains unclear. To investigate co-repressor activity in the context of this well defined signalling pathway, we have analysed the genome-wide binding profile of the best-characterized CSL co-repressor in *Drosophila*, Hairless, and of a second CSL interacting repressor, SMRTER. As predicted there was significant overlap between Hairless and its CSL DNA-binding partner, both in Kc cells and in wing discs, where they were predominantly found in chromatin with active enhancer marks. However, while the Hairless complex was widely present at some Notch regulated enhancers in the wing disc, no binding was detected at others, indicating that it is not essential for silencing *per se*. Further analysis of target enhancers confirmed differential requirements for Hairless. SMRTER binding significantly overlapped with Hairless, rather than complementing it, and many enhancers were apparently co-bound by both factors. Our analysis indicates that the actions of Hairless and SMRTER gate enhancers to Notch activity and to Ecdysone signalling respectively, to ensure that the appropriate levels and timing of target gene expression are achieved.

## Author summary

The communication between cells that occurs during development, as well as in disease contexts, involves a small number of signalling pathways of which the Notch pathway is one. One outstanding question is how these pathways can bring about different gene responses in different contexts. As gene expression is co-ordinated by a mixture of activators and repressors, we set out to investigate whether the distribution of repressors across the genome is important in shaping whether genes are able to respond to Notch activity. Our results from analyzing the binding profile of two repressors, Hairless and SMRTER, show that, in many cases, they are not essential for preventing a gene from responding. Instead they are deployed at a limited number of genetic loci where they gate the response, helping to set a threshold for gene activation. Perturbations to their function lead to enhanced gene expression in limited territories rather than to new programmes of gene expression. Their main role therefore is to restrict the time or levels of signal that a gene needs to receive before it will respond.

## Introduction

Growth, patterning and differentiation during development are coordinated by the activity of conserved signalling pathways whose action is largely realised through the transcriptional programmes they regulate. This necessitates mechanisms that ensure appropriate gene expression programmes are initiated, depending on cellular context, and that allow the fine tuning of gene expression in response to signalling levels. One way this is achieved is through the deployment of repressors **[1, 2] [3]**. Originally thought to be the primary factor that renders enhancers and promoters silent when signalling is absent, the role of repressors has become more enigmatic since several have been found to reside at sites of active chromatin **[4–6]**.

The Notch pathway is one example where the outcome of signalling is tuned by repressors. When activated, the Notch receptor becomes cleaved, releasing the intracellular domain, NICD, which collaborates with a DNA binding protein, CSL, to regulate gene expression **[7–9]**. Several different co-repressors have been found to interact directly with CSL and are proposed to keep target genes silenced in the absence of Notch activity **[10]**. In *Drosophila*, the best characterized co-repressor, Hairless, is a large unstructured protein that binds directly to Suppressor of Hairless (Su(H)), the *Drosophila* CSL **[11–15]**. As implied by their names, *Su(H)* was first identified on the basis of loss of function alleles that suppressed the phenotypes caused by a reduction in *Hairless* levels (*H/+*), highlighting the intimate relationship between these two proteins **[16, 17]**. Hairless itself functions as an adaptor, binding to Groucho and CtBP that in turn have the capability to interact with histone deacetylases (HDACs) **[18–20]**. It is thought that the HDACs prevent gene activity by modifying the local chromatin environment rendering it refractory to transcription.

While the model that CSL is complexed with repressors in the absence of Notch activity is a widely accepted one, there remain many uncertainties. First, although Hairless is a well established partner of Su(H) in *Drosophila*, it is not well conserved outside the Diptera **[21]** and its role appears to be fulfilled by multiple different proteins in mammals, including KyoT2 **[22, 23]**, MINT/SHARP **[24–26]** and SMRT **[27]**. While these proteins clearly bind to CSL and form a complex with HDACs and demethylases that modify chromatin **[25, 28]**, their functional roles have only been demonstrated at a few specific loci. Thus it remains questionable how widely such repression mechanisms operate. Second, with the diversity of CSL partners it is unclear what contributions the different types of complex might make, for example, whether they are recruited to different targets or have different sensitivities to NICD. Indeed, even in *Drosophila* the SMRT orthologue SMRTER, has also been reported to bind to CSL **[29]** and might contribute to repression at a subset of targets **[30, 31]**.

To gain insight into the regulatory contribution from CSL interacting co-repressors, we analysed the genome-wide binding profiles of Hairless in *Drosophila* Kc cells and in wing imaginal discs. As predicted, Hairless binding showed substantial overlap with that of Su(H), with a clear correlation in binding intensities. However, analysing the regulation of specific targets also revealed: (i) that the role of Hairless in silencing Notch targets is limited and is not a pre-requisite for a gene’s ability to respond to Notch activity; (ii) silencing of Hairless insensitive genes cannot be accounted for by binding of SMRTER, another co-repressor. The genome-wide profiles together revealed that many enhancers are bound by all 3 factors, Su(H), Hairless and SMRTER, and detailed investigations into an enhancer from *thread/Diap1* suggest this is indicative of co-regulation by Notch and Ecdysone signalling.

## Results

### Profile of Hairless occupancy in Kc cells shows recruitment to sites of active chromatin

To investigate the extent of Hairless recruitment to chromatin in Kc cells, two strategies were taken. First, GFP-tagged Hairless and GFP-Su(H) were generated in the context of genomic fragments spanning the gene loci, so that the proteins were expressed at close to physiological levels when introduced into Kc cells (e.g. S1 Fig). The chromatin association of GFP-Hairless in the Kc cells was then measured by chromatin immunoprecipitation (ChIP). With no DNA binding domain, Hairless is recruited to DNA indirectly and its binding was best detected using a two-step crosslinking method [6, 32]. Second, a Hairless-Dam fusion was generated, so that any sites of Hairless recruitment would become methylated [33]. Both methods yielded profiles with similar distributions across the genome (S1 Fig). We therefore generated a high confidence profile of Hairless occupancy by intersecting data from the two, although we note that the DamID data will be biased by the distribution of the target GATC sites. Over 50% of the regions identified by DamID were also detected by ChIP, a greater proportion than for similar data-sets from Groucho and GAGA-factor (S1 Fig [33]). This parsimonious approach identified 1406 Hairless bound regions (S1 Table). For comparison, a profile of GFP-Su(H) generated in a similar manner to GFP-Hairless, identified 376 bound regions (1% FDR, S1 Table).

**Fig 1:**
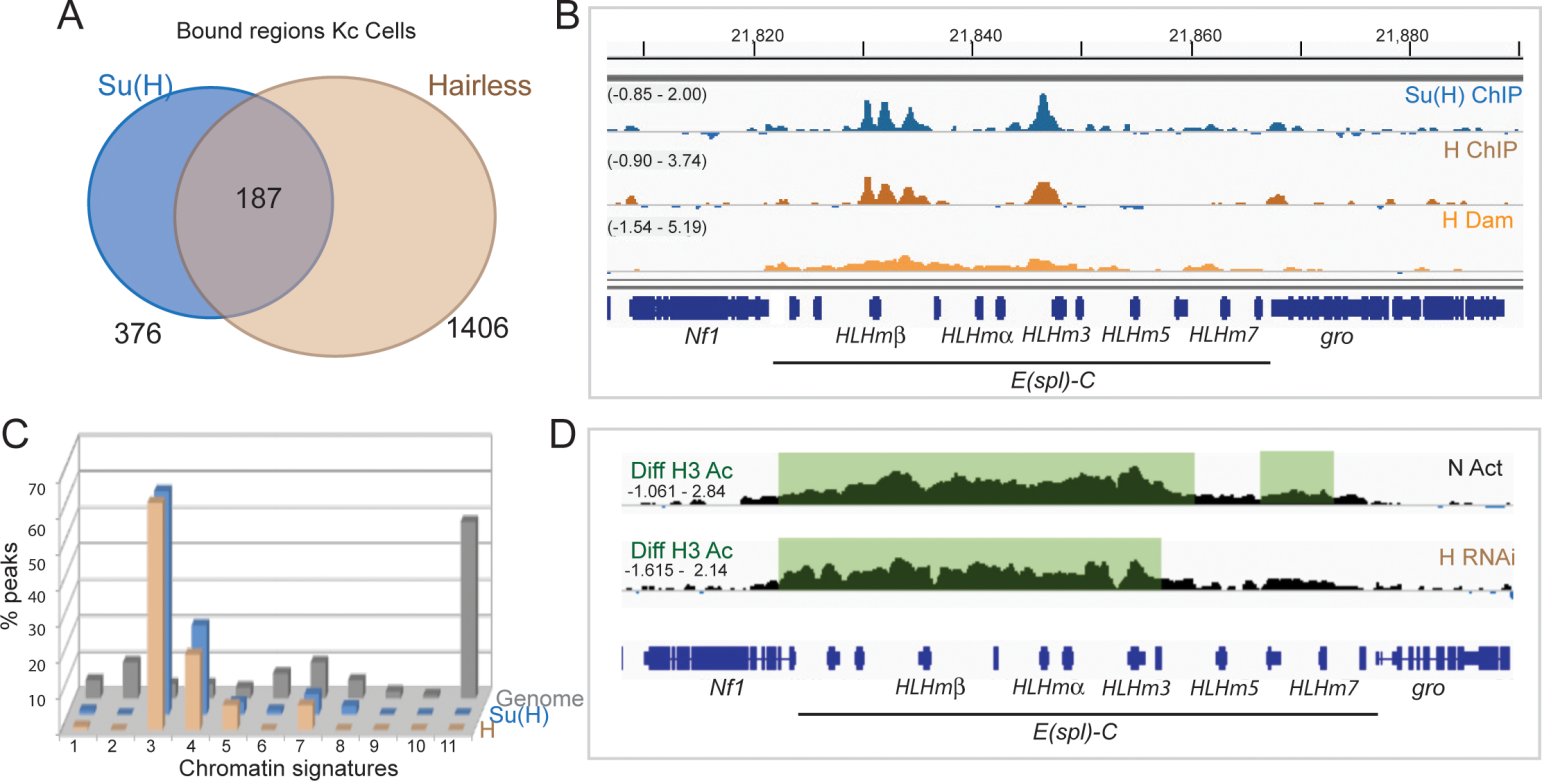
Hairless recruitment in Kc cells. (A) Venn diagram illustrating the proportion of Hairless bound regions in Kc cells that overlap with Su(H) binding. (B) Profile of Su(H) and Hairless across the *E(spl)* locus indicates co-binding. Graphs show GFP-Su(H) binding profile (blue graph: fold enrichment, Log_2_ scale is -0.85 to 2.00), (1% FDR); Hairless-GFP binding profile (brown: fold enrichment, Log_2_ scale is -0.90 to 3.74) and methylation enrichments from Hairless-Dam (orange: fold enrichment, Log_2_ scale is -1.54 to 5.19). Gene models are depicted in blue. (C) Distribution of Hairless occupied regions in relation to chromatin states, shows strong preference for signature 3, “enhancer” state (see methods and **[34]** for further details) (D) Knock-down of Hairless results in an increase in H3 acetylation similar to that seen with N activation (EGTA, 30 min). Graphs indicate differences in the enrichment profiles for H3K56ac ChIP from control and Notch activated (EGTA-treated) Kc cells or control and Hairless RNAi treated Kc cells, regions of significant difference are shaded (1% FDR; see **[34]**).

Su(H) is known to form a complex with Hairless **[11]** and, consistent with this model, the binding profiles of the proteins were found to overlap at many loci (Fig 1A and 1B). For example, at the well characterized Notch regulated *E(spl)*-complex genes, there was excellent correspondence of GFP-Su(H) and GFP-Hairless binding, while Hairless-Dam produced a broader profile centred on the same regions (Fig 1B). Overlapping the genome-wide binding profiles revealed that 50% of Su(H) bound regions corresponded with high confidence Hairless binding (Fig 1A). These co-occupied positions were also highly enriched for the Su(H) DNA binding motif (p = 3.36e-09). Furthermore, when the broader regions surrounding each Su(H) peak were considered, 83.5% had Hairless binding in the vicinity and in the majority (70%) of loci with Hairless and Su(H) binding more than one region of significant Hairless binding was detected. This is suggestive of extra contacts made through DNA looping or through the formation of large complexes.

Although many Su(H) bound regions exhibit Hairless binding in close proximity, the converse is not the case: 87% of regions enriched for Hairless do not overlap with Su(H) bound sites. There are two possible interpretations: one is that Su(H) binding at those positions was not captured (i.e. false negatives), the other is that additional factors besides Su(H) can recruit Hairless to DNA. Motif analysis suggests that the former explanation is likely to make, at best, a minor contribution, since enrichment for Su(H) motifs in the Hairless-only regions was only marginally significant (rank 407, p = 0.022). This suggests that other factors may contribute to Hairless recruitment. Many of the Hairless-only regions (>37%) also exhibit binding to the Hairless partner Groucho **[6]** (S2 Fig), consistent with these being *bona fide* co-repressor bound sites (e.g. CHES-1-Like). Finally, in cells depleted for Hairless, there were significant changes in RNA levels from genes that were bound by both Su(H) and Hairless as well as from those bound by Hairless only (S2 Fig). Notably however, the former were all increased, indicative of de-repression, whereas the Hairless-only genes exhibited both increased and decreased expression in Hairless depleted cells (S2 Fig;S3 Table). Two genes bound by Su(H) only were also deprepressed and are potentially examples of co-bound genes where we failed to detect Hairless binding (false negatives; S2 Fig; S3 Table).

While there may be different modes of recruitment, 63% of regions occupied by Hairless exhibited chromatin modifications associated with active enhancers (signature 3, Enh; Fig 1C; S2 Fig), characterized primarily by H3K4me1 and H3K27ac marks amongst others **[34]**. A smaller fraction (21%) mapped to regions with characteristics of active transcription start-sites (Signature 4, aTSS), which are also enriched in active chromatin marks. The remainder were located in intronic or primed chromatin. This distribution is broadly similar to that of Su(H) (Fig 1C; **[34])**, and the regions that were co-bound had similar profiles to those bound by Hairless only (S2 Fig). A slightly larger fraction of Su(H)-only regions were associated with active transcription start-sites (Signature 4/ aTSS; S2 Fig): a property also described for the mammalian homologue **[35]**. Given that Hairless containing complexes are proposed to repress transcription, the fact that it predominantly occupies active enhancer regions seems at first surprising. However, similar recruitment across active loci has also been observed for other co-repressors where, in some cases, they modulate the transcriptional response by curtailing the extent of active histone modifications **[4–6]**. To assess whether Hairless could do likewise, we examined the consequences of Hairless depletion on acetylated Histone H3 (H3K56ac), a modification that is increased following Notch activation **[34]**. We found no global changes in the levels of H3K56ac in Hairless depleted cells (S3 Fig). Strikingly however, there was a significant (1% FDR) increase at the *E(spl)* locus, similar to that observed when Notch signalling is activated, indicating that the presence of Hairless can suppress histone acetylation (Fig 1D). A small number (<10) of other loci also exhibited significantly increased H3K56ac (e.g. S3 Fig) while the remainder showed no change. Thus Hairless can suppress histone acetylation, but only at a subset of bound loci.

Taken together, these data support a model in which Hairless is recruited to enhancers by interactions with Su(H) and that it may, under some circumstances, modulate chromatin modifications at those positions and contribute to repression. However, the broad profile of Hairless binding indicates that it is also likely to be brought to chromatin independently of Su(H), and its effects may be complex as some bound regions exhibited increased gene expression in cells depleted of Hairless, whereas others showed decreases.

### Profile of Hairless occupancy in wing imaginal discs

To determine whether Hairless exhibits similar occupancy profiles in tissues, transgenic flies containing a genomic construct carrying GFP-tagged Hairless were generated. The line was capable of rescuing the viability of *H[P8]/H****[36]*** mutant flies, indicating that the GFP-Hairless protein was functional and expressed at physiological levels. The chromatin association of GFP-H in wing discs was determined by ChIP and compared to existing data for Su(H) occupancy **[37]**.

**Fig 2:**
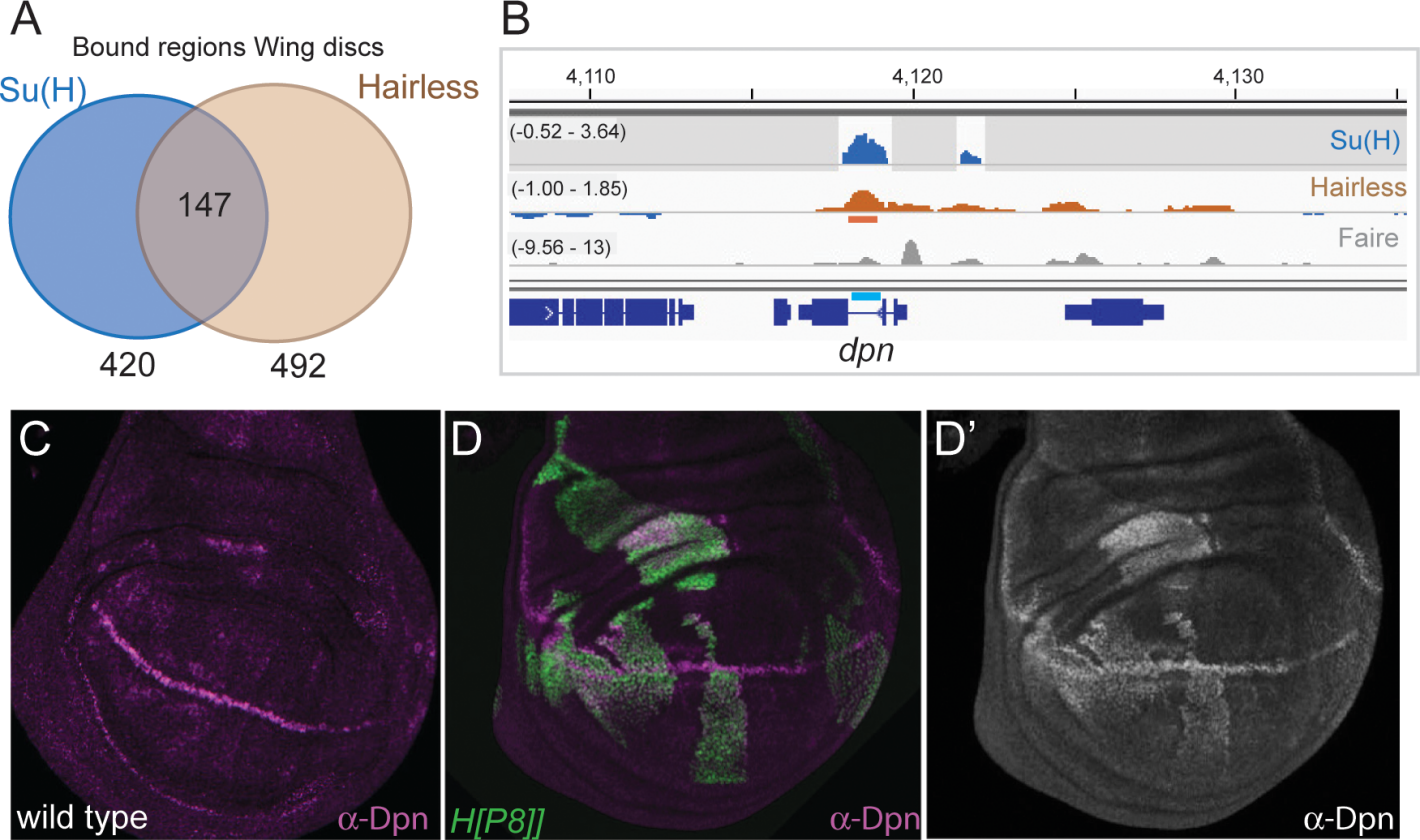
Hairless recruitment in wing imaginal discs. (**A**) Venn diagram illustrating the proportion of Hairless bound regions that overlap with Su(H) binding in wild-type discs. (**B**) Profile of Su(H) and H across the *dpn* locus indicates co-binding. Blue graph: regions of Su(H) binding (fold enrichment, Log_2_ scale -0.52 to 3.64; **[37]**). Brown graph: Hairless -GFP binding profile (fold enrichment, Log_2_ scale -1.00 to 1.85), horizontal lines below indicate regions of significant enrichment (peaks, 1% FDR). Grey graph: accessible chromatin identified by FAIRE **[63]**. Gene models are depicted in blue, location of identified wing-disc enhancer in cyan **[38]**. (**C**) Expression of *dpn* (purple) in wild-type wing disc, high levels of Dpn are detected at d/v boundary, lower levels in intervein regions. (D,D’) *dpn* (D, anti-Dpn, purple, D’, single channel white) is de-repressed in clones of cells with impaired Hairless (*H[P8]/H[P8]*, marked by GFP, green, D) at all locations in the wing disc.

Fewer regions (493; S4 Table) were detectably bound by Hairless in wing discs than in Kc cells, possibly because the profile represents the mean of binding in several different cell-types so that only regions bound in the majority of cells can be reliably identified. Nevertheless, a significant proportion (30%) of the 493 bound regions overlapped with Su(H) binding (Fig 2A), and were enriched for the Su(H) motif (p = 2.01e-6). Besides the well-characterized *E(spl)* locus, such regions included *deadpan* (*dpn*; Fig 2), *Serrate* and *Notch*. For example, both Su(H) and Hairless were bound at an intronic enhancer of the *deadpan* (*dpn*) gene that has been shown to confer expression in wing discs (Fig 2B; **[38]**. Consistent with the binding, we found that *dpn* expression was de-repressed throughout the wing-disc in clones of cells lacking Hairless (Fig 2C and 2D).

As with Kc cells, a significant proportion (70%) of the Hairless associated regions in wing discs did not overlap with the sites bound by Su(H). These Hairless-only regions were not enriched for the Su(H) motif (rank = 145 p = 0.045), suggesting that other factors are involved in Hairless recruitment. Indeed, a Hairless-GFP mutated in the Su(H) binding domain appears to still be recruited to chromatin, since bands indicative of binding to salivary gland polytene chromosomes are detected in live imaging experiments (S4 Fig). While one other partner for Hairless has been identified in embryos (Runt **[39]**), this is not expressed in wing discs. We examined the Hairless-only regions for binding motif enrichments and identified matches to Aef1 (p = 1.21e-24) and Grainyhead (p = 4.04e-10) motifs, which are both widely expressed in the wing disc and are proposed to act as repressors in at least some contexts, making them plausible Hairless partners **[40–42]**.

### Hairless binding is not detected at the cut or wg Notch-regulated enhancers

**Fig 3:**
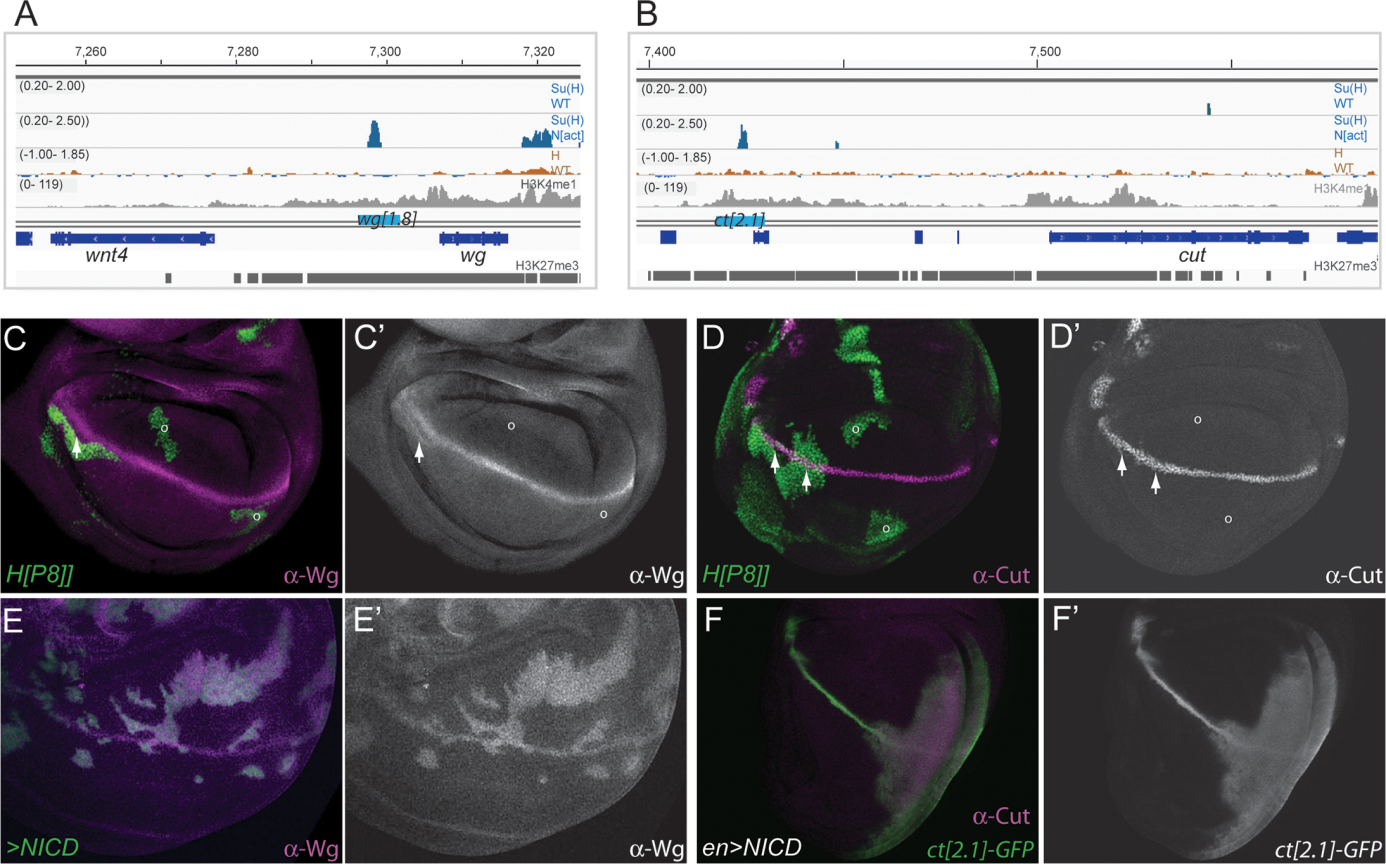
Hairless binding is not detected at the *cut* or *wingless* Notch responsive genes. **(A, B)** Profile of Su(H) and Hairless binding across genomic regions encompassing *wg* (A) and *cut* (B). Blue graphs: regions of Su(H) binding in control (Su(H) WT, fold enrichment, Log_2_ scale -0.20 to 2.00;) and Notch over-expression conditions (Su(H) N[act] fold enrichment, Log_2_ scale -0.20 to 2.50;**[37]**). Brown graph: Hairless-GFP binding profile (fold enrichment, Log_2_ scale -1.00 to 1.85). Grey graph: enrichment for H3K4me1 (scale: reads per million; **[64]**). Gene models are depicted in blue, identified wing-disc enhancers indicated by cyan bar above, and regions significantly enriched for H3K27me3 **[64]** indicated by grey bars below. (C, D) Removal of Hairless has limited effects on the expression of *wg* or *cut*. De-repression of *wg* (anti-Wg, purple, C; white C’) or *cut* (anti-Cut, purple, D; white D’) only occurs when cells with impaired Hairless (*H[P8]/H[P8]*, green, C,D) are close to the d/v boundary (arrows). No de-repression occurs at other locations (o). (E, F) Ectopic Notch activity elicits widespread activation of *wg* and *cut*. Ectopic expression of *wg* (anti-Wg, purple, E; white E’) occurs in clones of cells expressing NICD (green, E) at all locations. Expression of Cut (purple, F) and from a *cut* wing disc enhancer (*cut[2*.*1]-GFP*, green, F; white F’) occurs throughout the posterior compartment when NICD expression is driven by *en-Gal4*.

Two of the best-characterized Notch regulated genes in the wing disc are *cut* and *wingless* **[43–46]**. Both are normally expressed at high levels at the dorsoventral (D/V) boundary. However, they can be induced by ectopic Notch activity at other locations in the wing disc **[45]**. Strikingly, neither of these genes exhibited detectable binding of Hairless or Su(H) at their characterized wing-disc enhancers (Fig 3A and 3B), suggesting that these factors are not required to keep *cut* or *wg* silent at most positions in the disc. In agreement with this, and unlike the case of *dpn*, neither *cut* nor *wg* were generally de-repressed in Hairless mutant cells (Fig 3C and 3D). De-repression of these genes was only detected when clones were located close to the D/V boundary, the site where these genes are normally responsive (Fig 3C and 3D). At those positions, the loss of Hairless led to slight de-repression in some boundary-flanking cells (Fig 3C and 3D). Thus Hairless is likely to be recruited to these genes only in a limited subset of cells, where it is important to limit their expression, but is not required to repress their enhancers more broadly in the wing disc.

Both *wg* and *cut* could be induced much more widely when ectopic Notch was provided (Fig 3E and 3F; **[45]**, even though Hairless and Su(H) do not appear to occupy those loci in most cells. This suggests that stable binding of Su(H)/Hairless is not a pre-requisite to render the genes responsive. Indeed, in discs over-expressing NICD, both *cut* and *wg* enhancers showed significant enrichment for Su(H) binding (Fig 3A and 3B), indicating Su(H) can be recruited to those positions. Furthermore, reporters containing these enhancers exhibited the ability to respond widely to ectopic NICD (e.g. Fig 3F). Notably, the *cut* and *wg* enhancers are located in regions enriched for the H3K4me1 chromatin mark (Fig 3A and 3B), indicating that the chromatin is “primed”. Other Notch-regulated genes with similar characteristics included *scalloped* and *vestigial*, which are also enriched in H3K4me1 despite the lack of broadly detectable Su(H) occupancy.

### SMRTER binding shows limited overlap with Su(H)

**Fig 4:**
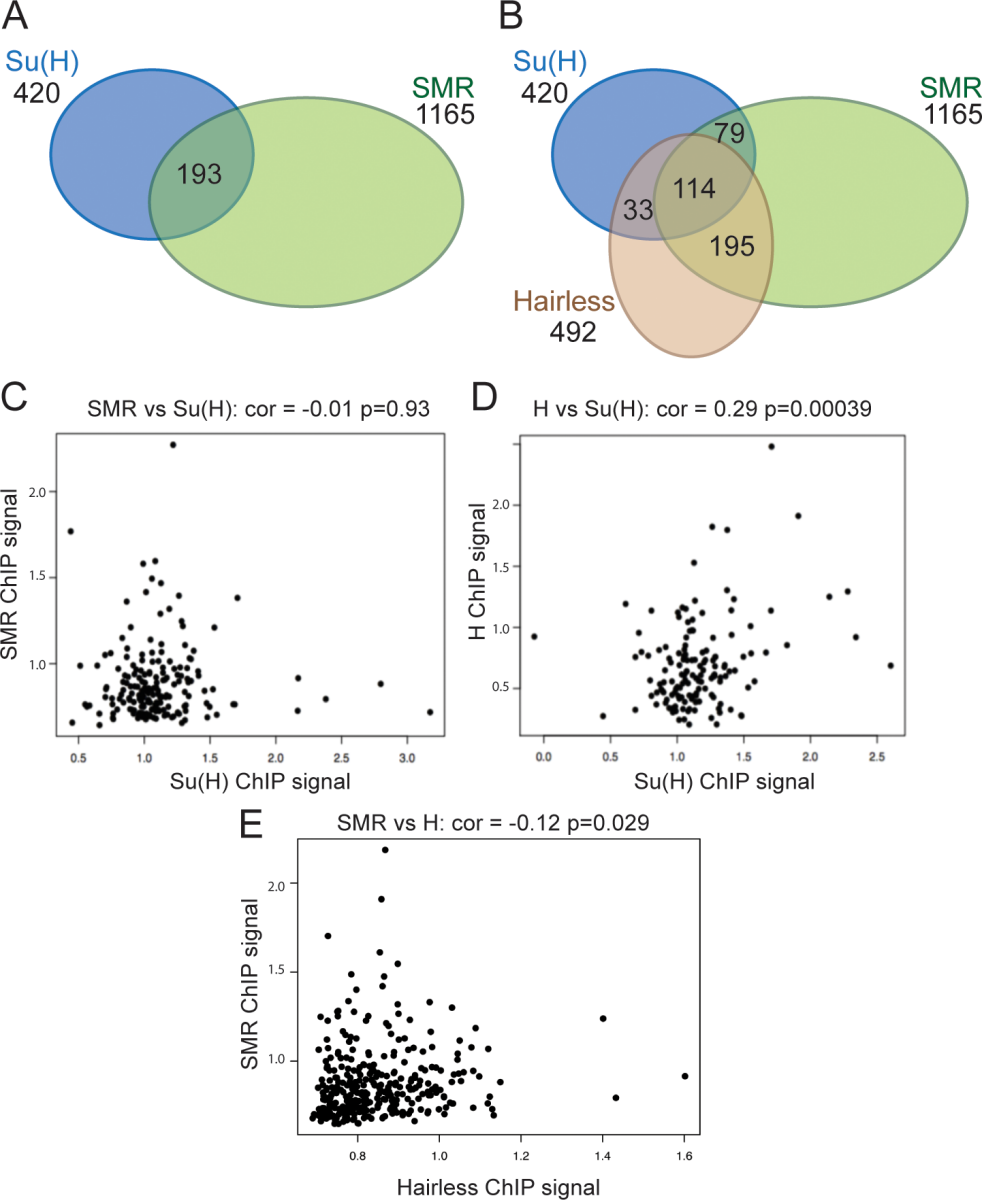
SMRTER binding overlaps with Su(H) and Hairless. (A, B) Venn diagrams illustrating the proportion of SMRTER (SMR) bound regions in Kc cells that overlap with Su(H) binding (A) and with both Su(H) and Hairless (B). (C-E) Correlations between the most highly enriched bound regions (enrichment, Log_2_ scale) for SMRTER and Su(H) (C), for Hairless and Su(H) (D) and for SMRTER and Hairless, (E). Only Hairless and Su(H) are very significantly correlated.

SMRTER is a co-repressor that, similar to Hairless, has been suggested to contribute to repression of Su(H)/Notch regulated targets **[30, 31]**. Since several loci did not exhibit Hairless binding, we reasoned that at least some of these might instead be dependent on SMRTER regulation. We therefore determined the genome-wide profile of SMRTER occupancy in wing discs, using an in-frame YFP fusion generated by a protein-trap transposon insertion **[47]**. We detected 1165 enriched regions (1% FDR; S5 Table), of which 16% overlapped with Su(H) bound regions (Fig 4A and 4B). This was similar to the number of Su(H) bound regions co-occupied by Hairless and included several genes potentially regulated by Notch, such as *thread/Diap1*, *E2f* and *anterior open* (*aop*). It is thus plausible that SMRTER could contribute to repression at Su(H) bound enhancers. However, no significant binding was detected at *dpn*, indicating that SMRTER is unlikely to be an obligate partner of Su(H)/Hairless, nor was binding detected at *cut* or *wg* enhancers.

Proteins that occupy the DNA together in a complex might be expected to exhibit similar enrichment profiles. Pairwise comparisons were therefore made of the top 100 bound regions, ranked by fold enrichment, for Su(H), SMRTER and Hairless. Only the profiles for Su(H) and Hairless exhibited a significant correlation (Fig 4D), no correlation was seen for Su(H) and SMRTER profiles (Fig 4C). However, the majority of Su(H) and SMRTER co-bound regions were also occupied by Hairless (Fig 4B). This “triple’ state was more frequent than would be expected by chance (p = 0.0004), suggesting that it is of significance and that these regions may be bound by more than one type of co-repressor complex.

### A thread/Diap1 enhancer is regulated by both Hairless and SMRTER

**Fig 5:**
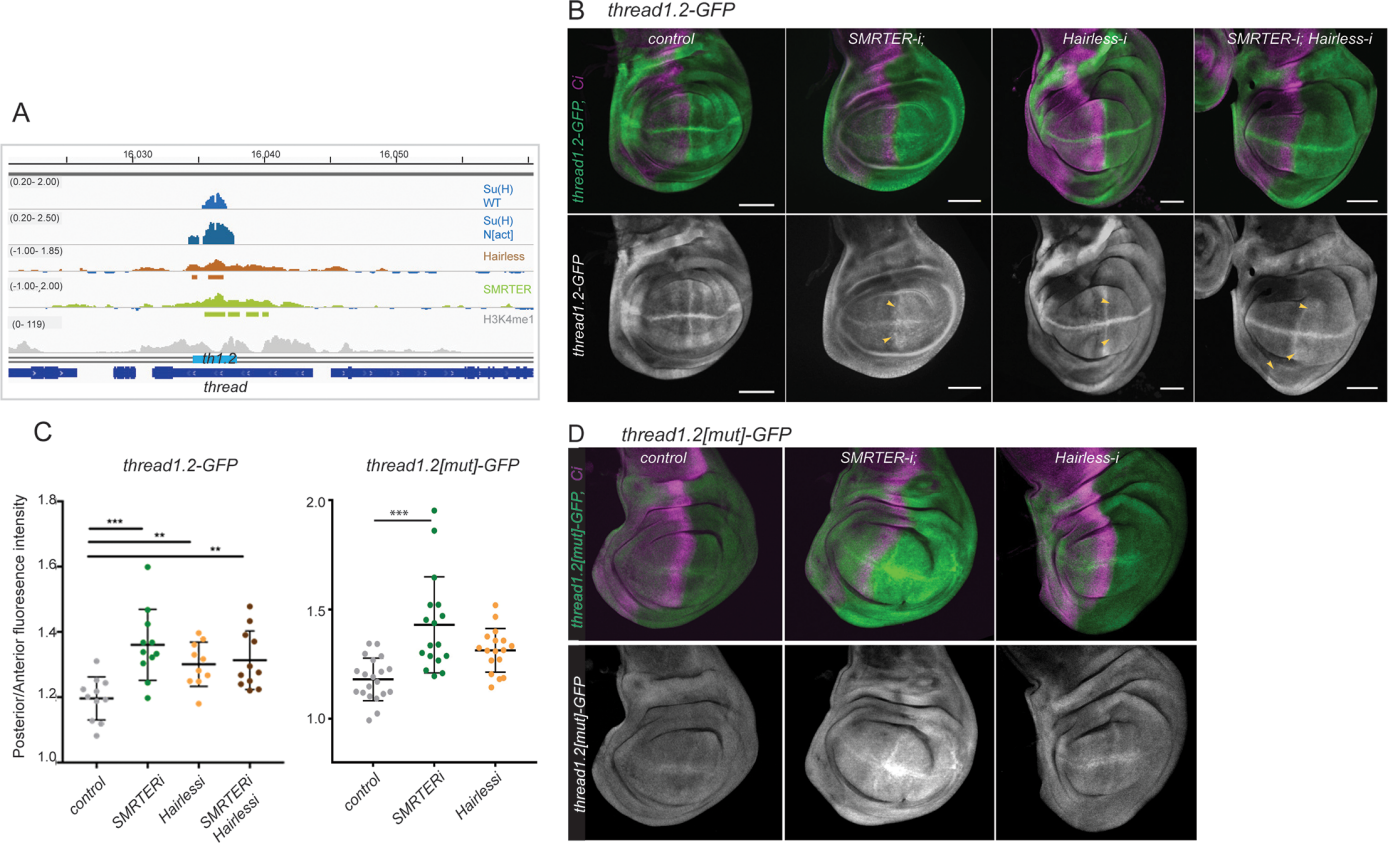
Both Hairless and SMRTER contribute to repression of *thread*. **(A)** Profile of Hairless and SMRTER binding across genomic region encompassing *thread/Diap1*. Blue graphs: regions of Su(H) binding in wing discs from control (Su(H) WT, fold enrichment, Log_2_ scale -0.20 to 2.00) and Notch over-expression conditions (Su(H) N[act] fold enrichment, Log_2_ scale -0.20 to 2.50) **[37]**). Brown graph: Hairless-GFP binding profile from wild-type wing discs (fold enrichment, Log_2_ scale -1.00 to 1.85), horizontal lines below indicate regions of significant enrichment (peaks, 1% FDR). Green graph: SMRTER-YFP binding profile from wild-type wing discs, (fold enrichment, Log_2_ scale -1.00 to 2.00), horizontal lines below indicate regions of significant enrichment (peaks, 1% FDR). Grey graph: enrichment for H3K4me1 (scale: reads per million; **[64]**). Gene models are depicted in blue, an identified wing-disc enhancer (*thread1*.*2*) is indicated by the cyan bar. **(B)** Depletion of Hairless or SMRTER leads to de-repression of a *thread1*.*2-GFP* reporter (green, upper; white, lower, yellow arrowheads). Wing discs expressing the indicated RNAi in the e*ngrailed* domain, complementary to the regions detected by anti-Ci (where necessary, *UAS-w-RNAi* or *UAS-GFP-RNAi* were included so that all genotypes contained the same number of UAS insertions; see methods). (C) Quantitation of the changes in expression of *thread1*.*2* (left) and *thread1*.*2[mut]* (right) reporters in the indicated genotypes, expressed as ratio of fluorescence intensities in posterior (manipulated) versus anterior (un-manipulated) territories. (D) Mutated *thread* enhancer, *thread1*.*2[mut]* remains sensitive to depletion of SMRTER but not Hairless, conditions as in B. In B and D, anti-Ci (purple) marks un-manipulated anterior territory.

One region that was enriched for SMRTER, Su(H) and Hairless binding was within an intron of *thread/Diap1* (Fig 5A*)*. This region exhibited enhancer activity, conferring a similar expression pattern to the endogenous gene when placed upstream of a GFP reporter (*th1.2-GFP*; Fig 5B; **[37]**). We therefore examined the effects on the expression of this reporter when depleting Hairless and SMRTER, by targeting RNAi to the posterior part of the wing-disc (using *engrailed-Gal4 Gal80ts*). Knock down of either of the repressors resulted in de-repression of *th1.2-GFP* (Fig 5B and 5C). In the case of SMRTER depletion, the resulting reporter pattern was still highly modulated whereas with Hairless depletion the expression was more uniform (Fig 5B). When both SMRTER and Hairless were knocked-down together we observed an additive effect on *th1*.*2-GFP*, although the overall expression levels were not significantly elevated in the double combination (Fig 5B and 5C). Similar effects were seen with enhancers from the *reaper* and *cut* genes, which were de-repressed when Hairless or SMRTER were depleted (S5 Fig).

To investigate whether the functions of Hairless and/or SMRTER are mediated through Su(H), the Su(H) binding motif in the *th1*.*2* enhancer was mutated (*th1*.*2[mut]*) and its expression analysed in similar knock-down experiments. Under wild type conditions, low levels of residual expression were observed with the mutated enhancer, which were little changed by the knock-down of Hairless (Fig 5C and 5D). In contrast, SMRTER knock-down resulted in a significant de-repression (Fig 5C and 5D). This implies that SMRTER contributes to the repression of the *th1*.*2* enhancer, but that it does so independent of Su(H).

### SMRTER binding correlates with Ecdysone receptor

**Fig 6:**
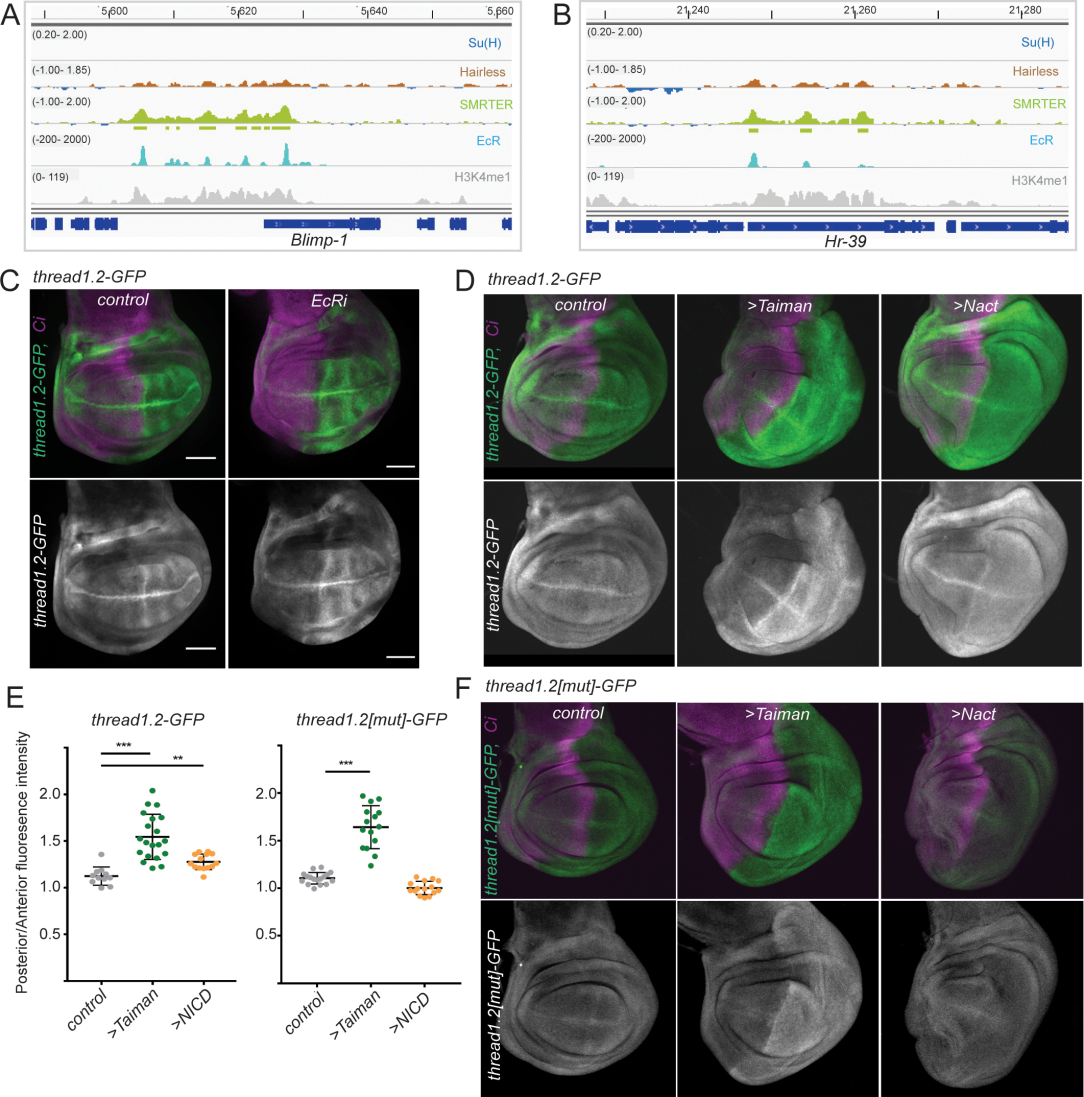
**SMRTER binding is indicative of co-regulation by EcR (A, B)** Profile of Hairless, SMRTER and EcR binding across genomic regions encompassing *Blimp-1* (A) and *HR39C* (B). Blue, brown green and grey graphs as summarized in Fig 5A. Cyan graph: Enrichment for EcR binding in wing discs from 0–4 hour white pre-pupae **[48]**. (C) Depletion of EcR leads to de-repression of *th1*.*2-GFP* (green, upper; white, lower). Wing discs expressing *EcR RNAi* driven by *en-Gal4* (complementary to the domain of Ci, purple; where necessary *UAS-w-RNAi* or *UAS-GFP-RNAi* were included so that all genotypes contained the same number of UAS insertions; see methods). (D) Widespread *thread1*.*2-GFP* expression throughout the domain of *en-Gal4* where ectopic Taiman or NICD are driven (complimentary to the regions detected by Ci, purple). (E) Quantitation of the changes in expression of *thread1*.*2*-GFP (left) and *thread1*.*2[mut]-GFP* (right) reporters in the indicated genotypes, expressed as ratio of fluorescence intensities in posterior (manipulated) versus anterior (un-manipulated) territories. (F) Mutated *thread* enhancer, *thread1*.*2[mut]* remains sensitive to expression of Taiman but not NICD, conditions as in D.

Since SMRTER can exert repression at an enhancer that lacks a Su(H) binding motif, it is likely that it is recruited to chromatin by another DNA binding protein. In this respect, SMRTER has been proposed to act as a co-repressor for the Ecdysone receptor (EcR; **[29, 30]**) and we found that SMRTER binding peaks were significantly enriched for one of the EcR motifs (EcR::USP, p = 5.2e-7). We therefore compared the profile of SMRTER binding to published EcR binding data from a similar stage **[48]**. 38% of SMRTER peaks overlapped with EcR bound regions and there was a significant correlation between the SMRTER and EcR peaks when the top 100 peaks (ranked by enrichment) were compared (cor = 0.19; p = 1.5e-05). Target genes that were highly enriched for SMRTER and EcR binding included *Hr39* and *Blimp-1* (Fig 6A and 6B).

We examined whether EcR was involved in the regulation of the *th1*.*2-GFP* reporter by analysing the effects of EcR depletion, which should mimic those observed with SMRTER depletion **[29, 49]**, and of expressing the EcR co-activator, Taiman **[50]**. As predicted, EcR depletion resulted in a mild increase in *th1*.*2GFP* expression, similar to SMRTER knock-down (Fig 6C), while Taiman expression resulted in high levels of uniform *th1*.*2GFP* expression, as did expression of NICD (Fig 6D and 6E). The up-regulation induced by Taiman was independent of the presence of the Su(H) motif, as a similar response was detected with the mutated *th1*.*2[mut]* enhancer, while the response to NICD was abolished (Fig 6E and 6F). These data therefore support a model where SMRTER is recruited to the *th1*.*2* enhancer through EcR and suggest that the co-occurrence of SMRTER, Hairless and Su(H) is likely indicative of enhancers that are co-regulated by Ecdysone and Notch.

## Discussion

In prevailing models for Notch mediated regulation of target genes, target loci are bound by a CSL-co-repressor complex, from which the co-repressors are displaced by NICD to activate transcription **[8, 10]**. Our data support the notion that the co-repressor Hairless is bound with Su(H) at several Notch regulated enhancers, since their genome-wide binding profiles in both Kc cells and wing discs exhibited considerable overlaps. Furthermore, depletion of Hairless in Kc cells resulted in transcriptional de-repression and an increase in histone acetylation at some loci. Likewise, the absence of Hairless led to de-repression of target-genes such as *deadpan* and *thread/Diap1* throughout the wing disc.

Our results, however, challenge the view that pre-binding of the Su(H)/Hairless repressor complex is required for genes to be able to respond to NICD. Several well-known Notch regulated genes, such as *cut*, *wg* and *vg* did not generally display Hairless (or Su(H)) binding in the wing disc despite the fact that they are strongly induced throughout the disc when ectopic NICD is provided. This implies that their silencing is not generally dependant on the Hairless/Su(H) repressor complex. In agreement with this idea, no de-repression of *cut* or *wg* occurred in most cells lacking *Hairless* and only a very modest de-repression was observed at the dorsoventral boundary where these genes are normally expressed. It is therefore likely that Hairless is recruited in only a small population of cells where it may help to dampen or refine the response of target enhancers. This is borne out by the limited number of genes that are de-repressed by knock-down of *Hairless* in Kc cells.

Since relatively few Notch regulated genes were bound by Hairless, this raised the possibility that an alternative co-repressor might be recruited at other loci. For example, one of the CSL partners in mammalian cells is SMRT **[27]**, whose homologue SMRTER was found to co-IP with Su(H) in flies **[31]**, making it a plausible candidate **[30]**. Analysis of genome-wide SMRTER binding indicated that a significant fraction of Su(H) bound regions were also occupied by SMRTER, although the binding intensities were not well correlated. However, many of the co-bound sites were also regions enriched for Hairless, including *thread/diap1*, which was robustly enriched for all 3 proteins. Further analysis of *thread* regulation demonstrated that both Hairless and SMRTER are important for suppressing its expression, albeit in slightly different ways. When the Su(H) motifs in the *thread* enhancer were mutated, only the Hairless regulation was lost while that of SMRTER remained. These data, along with the strong correlation between SMRTER and Ecdysone Receptor binding, imply that the co-repressors are recruited to the same enhancer via different DNA-binding partners: Hairless via Su(H) and SMRTER via EcR. Crosstalk between Notch and EcR also occurs at the *cut* enhancer, although this involves Broad rather than EcR **[51]**. Thus we conclude that much of the co-localization of SMRTER and Su(H) is likely indicative of genes that are regulated by inputs from the EcR and Notch pathways, although we cannot fully rule out the possibility that SMRTER could act via Su(H) in some circumstances.

A striking feature of the Hairless bound regions is that they correlate with regions of active chromatin, despite Hairless involvement in repression. Similar features have been noted for Groucho **[6]**, a key partner of Hairless, and for another class of repressors/co-repressors **[5]**. Likewise, Dorsal repressed enhancers exhibited some active chromatin marks (H3K4me1) although these were found to be hypoacetylated compared to their active counterparts **[52, 53]**. It is proposed these repressive factors contribute to polymerase pausing **[6, 52]** rather than more long-term silencing **[53]**, so that gene transcription would be kept in check until/unless conditions change and RNA polymerase is released. Another suggestion is that modulation by repressors enables a fine regulatory control over transcriptional output from a given target gene so that it can be graded over range of values. Indeed, the mammalian CSL co-repressor SHARP has been suggested to control a permissive chromatin state at Notch target genes by concomitantly promoting H3K27 deacetylation and H3K4 methylation (the former would dampen activity while the latter would enhance it; **[54]**). The modest changes in acetylated histones that occurred at some highly regulated loci in Hairless depleted cells would fit with these models, as would the varied changes in gene expression that occur in the Kc cells. Thus we propose that Hairless, like SHARP and other similar factors, is likely to interact with a range of partners through which it will modulate, rather than silence, the response of a target enhancer to the levels of Notch activity, most likely via local effects on chromatin.

## Materials and methods

### Hairless-GFP Su(H)-GFP and Hairless-Dam constructs

To generate a genomic Hairless construct, AttB plasmids containing the genomic region 3R: 20621141–20628985 (using primers 5’-GCATTCGTCTCAATAACTAACGTCG 5’-CGCAATAAAAAGACACCTGCAACC) were constructed, with the coding sequences of eGFP (enhanced Green Fluorescent Protein) and Dam methylase **[33]** inserted in-frame before the stop codon located at exon 4, to generate a protein fusion at the C-terminus. This plasmid was used in transfection experiments (see below) and subsequently injected into strains containing an AttP40 site to generate transgenic Hairless-GFP flies. The functionality was assessed by its ability to rescue *H[P8]/H[1]* flies; the viability of the mutant flies was rescued (although they were not fully fertile) indicating that the plasmid confers functional Hairless activity.

For the genomic GFP-Su(H), an AttB plasmid containing the genomic region 2L 15038840–15045039 (using primers 5’ -CAAGTTAGATATGGCAATGCACCG 5’-ACTGCATATCTGTACTGATGACG) was constructed, with the coding sequences of eGFP inserted in frame at the start of exon 1, to generate a protein fusion at the N-terminus. This plasmid was used in transfection experiments (see below).

### Cell culture conditions

Kc cells were cultured at 25 ^O^C in Shields and Sang M3 insect medium (Sigma, S3652), supplemented with 5% FBS (Sigma, F9665), 1g/L yeast extract (Oxoid, LP0021), 2.5g/L bacto-peptone (BD Biosciences, 211677) and 1x Antibiotic-Antimycotic (Gibco, 15240–062). For *Hairless* RNAi, Kc cells were transfected with 20μg dsRNA in Fugene 6 (Promega, E2691) in 10 cm plates according to the standard protocol and then incubated for 72h. RNA isolation was performed using Qiagen Rneasy Midi kit (cat N 75142).

### Labelling and hybridisation to expression arrays

To 50μg total RNA in 28μl DEPC water 1μl of 500 ng/μl oligo (dT)23 anchored primer (Sigma) was added and incubated at 65°C for 10 minutes, then placed on ice. 8μl of 5x first strand buffer (Invitrogen), 2μl of low-C dNTP mix (5mM dATP, dGTP, dTTP, 2mM dCTP), 2μl of 1mM Cy3 or Cy5 dCTP (GE Healthcare), 2μl of 0.1M DTT (Invitrogen), 0.5μl of RNAsin (Promega) and 2μl of Superscript III reverse transcriptase (Invitrogen) were added and incubated at 46°C for 2 hours. The reaction was stopped with 20μl mix of 0.5M EDTA and 1M NaOH and incubated at 65°C for 15 minutes. Sample was neutralised with 25μl of 1M Tris-HCl (pH 7.5). Combined Cy3- and Cy5-labelled probe (sample and control) were purified with AutoSeq G-50 column (GE Healthcare) and the volume reduced to between 2-5μl in a speed vac with medium heat. Then 2μl of 10mg/ml sonicated salmon sperm DNA (Invitrogen) and 140μl of Ocimum hybridisation buffer were added to the labelled mixture and boiled at 100°C for 2min. 140μl of the labelled sample was loaded to 70-mer long oligo microarrays (FL003, Flychip), and hybridised for 16 hours at 51°C. Post-hybridisation washes were performed as per PowerMatrix slides protocol (FMB). Arrays were scanned at 5μm resolution with a GenePix 4000B (Axon) dual laser scanner. Images were processed and spot quantified by the Dapple software (http://www.cs.wustl.edu/~jbuhler/research/dapple/). Raw data were then loaded into limma (Bioconductor; **[55]**) and normalised with the vsn package (Bioconductor; **[56]**). The resulting ratios are log2 ratio of sample/control. Four biological replicates were analysed, data are available from GEO as part of super series GSE97603. Genes with altered expression were assigned to a ChIP peak(s) if the peak was within 10kb (upstream or downstream).

### Chromatin immunoprecipitation and DamID from Kc cells

For Chromatin immunopreciptation Kc cells were transfected with Hairless-GFP or GFP-Su(H) plasmids in combination with a plasmid containing puromicyn resistance gene (pMT-Puro) using Fugene 6 and grown under puromycin selection (2μg/ml). Chromatin preparation and ChIP were essentially as described previously **[34] [57]**, except the cells were fixed in 1% formaldehyde, 1mM EGS for 15min. A library was generated from the immunoprecipitated DNA using a complete whole genome amplification (WGA) Kit (GenomePlex #WGA2-50RXN). The amplification reaction was supplemented by adding 0.75ul of 10mM dUTP. The amplification was performed for 14 cycles. After purification with Qiagen PCR cleanup columns, 7.5μg of purified DNA was hybridized to GeneChip Drosophila Tiling 2.0R Array according to Affymetrix specifications. Three independent replicates were performed for each construct.

For DamID experiments, KC cells were transfected with Hairless–Dam using Fugene 6 and incubated for 72 hours before harvesting. Genomic DNA was extracted using Qiagen DNeasy Blood and Tissue Kit (#69504 or #69506), following manufacturer's instructions. 2.5μg of genomic DNA were digested in 10μl with *Dpn*I overnight at 37°C. *Dpn*I was inactivated at 80°C for 20min. Genomic DNA fragments were ligated to Phosphorylated AdR adapters (AdRt 5' CTAATACGACTCACTATAGGGCAGCGTGGTCGCGGCCGAGGA, aligned to AdRb 5' TCCTCGGCCG) in 20μl reaction for 2 hours at 22°C using FERMENTAS (cat# EL0011), supplemented with PEG-4000. The reaction was inactivated at 65°C for 10 min and subsequently digested with *Dpn*II, the resulting DNA fragments were cleaned using Qiagen PCR cleaning kit (Cat#28104). DNA was amplified for 15 cycles by PCR using Expand DNA polymerase (Cat#11732641001) supplemented with 0.1mM dUTP and Adr-PCR primer (5' GGTCGCGGCCGAGGATC). After amplification, adapters were removed by *Dpn*II digestion and cleaned using Qiagen PCR cleaning columns. Samples were then fragmented and hybridized to GeneChip Drosophila Tiling 2.0R Array according to Affymetrix specifications. Three replicates of the Hairless-Dam and a Dam-only control were performed.

### Chromatin immunoprecipitation from wing imaginal discs

Dissected heads from 30 third instar larvae (Hairless-GFP or SMRTER-YFP) were fixed with 1ml of 4% Formaldehyde +1.5mM of EGS (25mins at room temperature) and the reaction quenched with 200μl of 2M-glycine solution. After rinsing 3x (1 x PBS + Roche complete protease inhibitor cocktail) the wing discs were removed and transferred to 50μl of nuclear lysis buffer (50 mM Tris-HCl pH 8; 10 mM EDTA; 1% SDS; 1x Roche complete protease inhibitor) for 10 min on ice before homogenising the wing discs. 250μl of IPDB (ImmunoPrecipitation Dilution Buffer) was then added to the mixture and the sample was sonicated for 7 min, with a 30 second “ON/OFF” cycle, in a pre-cooled Bioruptor (Diagenode UCD-200). After sonication, the sample was centrifuged at 13200rpm at 4°C for 10 min, and the chromatin containing supernatant then diluted with 200μl of IPDB. ChIP was performed as previously described **[37, 57]**, using polyclonal rabbit anti-GFP (Abcam ab290). Three ChIP samples were then pooled for library preparation using the GenomePlex Complete WGA kit. 14 rounds of amplification were performed and the DNA purified using Qiagen PCR cleanup columns. 250μg DNA was then subjected to a further 10 rounds of amplification in the presence of 0.75μl of 10mM dUTP. After purification with Qiagen PCR cleanup columns approximately 7.5μg of DNA was hybridised to Affymetrix GeneChip Drosophila Tiling 2.0R Arrays according to Affymetrix specifications. Three (Hairless) or two (SMRTER) independent replicate experiments were performed.

### ChIP data analysis

Window smoothing and peak calling were performed using the Bioconductor package Ringo **[58]** with a winHalfSize of 700bp and min.probes = 10. Probe levels were then assigned p-values based on the normalNull method, corrected for multiple testing using the Hochberg-Benjamini algorithm and then condensed into regions using distCutOff of 200bp. All data are available from GEO, super-series accession number GSE97603.

Dataset overlaps were analysed using GenomicRanges (Bioconductor R; **[59]**) and for overlap with chromatin signatures, the profile for Kc cells was used **[34]** (https://github.com/rstojnic/notch-chromatin). Motif enrichment analysis was performed using the Bioconductor package PWMEnrich **[60]**, which assesses the enrichment of each motif from a library of 650 experimentally derived DNA motifs for *Drosophila* transcription factors. Accessible chromatin in BG3 and Kc167 cells was used as a background for calculating P-values of enrichment. The correlation between ChIP signals was calculated on enhancers that are a union of all ChIP peaks from the considered datasets. The average signal over the whole enhancer was then plotted for pairs of datasets and the Pearson correlation and corresponding P-value calculated.

Analysis of K56 acetylation changes in Kc cells following *Hairless* RNAi was carried out using ChIP-chip with the conditions and methods described previously **[34]**. For controls, cells were treated with GFP RNAi. To identify regions of differential K56 acetylation an algorithm was used to compare the variance between replicates to the variance between samples within sliding 2kb regions **[34]**. This method identifies regions where the differences cannot be explained by noise, using two replicates for each sample (e.g. con1, con2 to HRNAi1, HRNAi2 where 1,2 indicate replicates). The noise distribution was used to convert differences between con and HRNAi within individual 2kb regions into a P-value, which was then corrected for multiple testing using the Hochberg-Benjamini algorithm. The resulting P-value represents the False Discovery Rate (FDR). An FDR threshold of 1% was used to define regions of significant difference.

The significance calculation of combinatorial overlaps strongly depends on the number of unoccupied enhancers **[61]**, and the binding model used. We used a null model in which Su(H)-H binding is dependent on each other (as estimated from available binding data), but SMRTER binding is independent of both. We assumed there is between 0 and 5000 additional sites in the genome that are unoccupied but that could in principle be bound. We inferred the number of unoccupied enhancers by maximising the likelihood of the fit. Finally, we calculated the P-value of the difference between the observed and expected individual patterns using Fisher’s exact test. We found that 70% more enhancers are bound by all three factors (Su(H), H, SMRTER) than expected by our best-fitting model (P-value 0.0004).

### Drosophila genetics and immunofluorescence

All alleles and stocks are described in FlyBase (www.flybase.org) unless otherwise indicated. The following mutant and reporter lines were used: *H[P8]*, H[P1] *SMARTER-YFP[CPTI001385]*; and *th1*.*2-GFP* **[37]**; *cut1*.*3-GFP* and *wg2*.*2-GFP*. In RNAi and overexpression experiments, the Gal4 driver stock *engrailed*:*Gal4 Tub*:*Gal80*^*ts*^ was combined with UAS lines and larvae were shifted to 30°C 48 hours after egg laying. The following RNAi and overexpression lines were used: *UAS-w RNAi (*TRiPGL00094), *UAS-Hairless RNAi (*TRiPJF02624), *UAS-SMRTER RNAi (*KK1026110), *UAS-Notch-intra[79*.*2]; UAS-Taiman* **[50]**; *UAS-EcR-RNAi*; *UAS-GFP-RNAi* (BL-9330); *UAS-lacZ*. Note that *UAS-wRNAi*, *UAS-GFP RNAi* or *UAS-lacZ* were included where appropriate to ensure the number of UAS sequences was kept consistent in each experiment.

To generate MARCM clones **[62]** lacking Hairless, *H[P8] FRT82B/TM6B* were crossed to *hs-FLP tubGal4 UAS-GFP; FRT82B tubGal80*. Progeny were heat shocked at 37°C for 1 hour, 72 hours after egg-laying then kept at 30°C until dissection.

Fixation and immunostaining conditions were as previously described. Antibodies used were: rat anti-Ci (DSHB 2A1; 1/25), mouse anti-Cut (DSHB 2B10; 1/20), mouse anti-Wg (DSHB 4D4; 1/20), guinea-pig anti-Dpn (gift from Christos Delidakis; 1/50), rabbit anti-GFP (Invitrogen; 1/2000). Alexa conjugated secondary antibodies were from ThermoFisher.

Fluorescence quantifications were performed using ImageJ64. Identical laser and confocal settings were used throughout a single experiment, and in all cases measurements were from at least two independent experiments. An experiment-specific-ROI (Region Of Interest) was pre-defined using ImageJ64, and, for a given experiment, consistent ROI settings were used across genotypes and they were placed at similar positions. Mean grey value measurements for the ROI within the manipulated posterior region were normalised to measurements for the ROI in the wild type anterior compartment for each wing disc. Individual data points are the relative values for each wing disc analysed, and the mean value was calculated for comparison between genotypes. The significance of differences in the expression ratios was assessed using a T-test; p<0.05 is indicated by single asterisk, p<0.01 is indicated by double asterisk and p<0.001 is indicated by triple asterisk.

## Supporting information

S1 Table High confidence sites of Hairless binding in Kc cells. Binding intervals defined by overlap between regions significantly methylated by Hairless-Dam and enriched in Hairless-GFP ChIP.
(XLSX)

S2 Table Regions bound by Su(H)-GFP in Kc cells (1% FDR). (XLSX)

S3 Table RNAs with fold change in expression following Hairless RNAi in Kc cells (p<0.05). (XLSX)

S4 Table Regions bound by Hairless-GFP in wing discs (1% FDR). (XLSX)

S5 Table Regions bound by SMR-YFP in wing discs (1% FDR). (XLSX)

S1 Fig Relationship between Hairless-GFP ChIP and DamID profiles in Kc cells. (A) Expression levels from plasmids with tagged genomic fragments are similar to endogenous levels. Western blot detecting GFP from controls (no Su(H)-GFP) and from Kc cells containing GFP-Su(H) or a control plasmid. A larger band corresponding to GFP-Su(H) (large arrow) is detected in cells containing the Su(H)-GFP plasmid, expression levels are similar to endogenous Su(H) (small arrows) present in all extracts. (B) Venn diagram illustrating overlap between regions significantly methylated by Hairless-Dam (10% FDR; pale orange) and enriched in Hairless-GFP ChIP (1% FDR; brown). The overlap is highly significant (p = 0, Fishers exact test). Inset; Overlap between similar data sets for Groucho from **[6]**. (C) Genomic region from 2L comparing the profiles of replicates (orange) from H-DamID (upper, R1-R3) and H-GFP ChIP (lower R1-R3) with the combined profile obtained for each dataset (brown). Fold enrichment, Log_2_ scale -0.50 to -1.5. Orange bars indicate regions enriched in both H-GFP ChIP and H-DamID, referred to as Hairless high confidence bound regions. Gene models are depicted in blue. (D) Percentage of peaks from the different data sets as indicated overlapping with the genomic features, coloured according to the legend.
(TIF)

S2 Fig Characteristics of regions bound by Su(H) and/or Hairless-in Kc cells. (A) Distributions of regions occupied by Su(H)+Hairless, Su(H) only or Hairless only in relation to chromatin states, all exhibit strong preference for signature 3, “enhancer” state and, to a lesser extent, signature 4 active TSS state (see methods and **[34]** for further details). (B) Percentage of peaks from the different data sub-sets as indicated overlapping with the genomic features, coloured according to the legend. (C) Percentage of peaks from the different indicated datasets overlapping with the regions bound by Groucho (from **[6]**). (D) Fold change in RNA expression levels when Hairless is depleted for genes associated with the indicated subsets of bound regions. Left graph depicts all genes with significant change in expression (p≤0.5); Right graph, only those genes with log2 fold change >0.4 (p≤0.05).
(TIF)

S3 Fig Effects of Hairless depletion on H3K56 acetylation in Kc cells. (A-B) Empirical distribution frequency plots comparing K56ac enrichment in control (GFP-RNAi) and Hairless depleted (H-RNAi) cells at all peaks and at Hairless peaks, no large-scale differences are detected. (C) Empirical distribution frequency plot comparing differential K56ac levels at H peaks (red) and Su(H) peaks, indicates that they are similar, with Su(H) peaks showing a slightly higher frequency of changes. (D) Knock-down of H results in an increase in H3 acetylation at a few H bound positions, illustrated by *hh*. Graphs show H3K56ac enrichment (brown) and differences in H3K56ac enrichment (green) in control and H RNAi treated Kc cells, regions of significant difference are indicated by the green box (1% FDR; see **[34]**). Hairless bound regions are indicated by blue boxes; not all H bound regions exhibit significant changes in H3K56ac when Hairless is depleted.
(TIF)

S4 Fig Detecting chromatin association by live imaging of Hairless-GFP in salivary gland nuclei. Both wildtype and Hairless L235D (a mutation that perturbs Su(H) binding, [11] are seen to form bright “bands” (e.g orange arrows) indicative of binding to the polytene nuclei, in unfixed salivary gland nuclei. (A) Projections of focal sections through nuclei of the indicated genotypes where parts of chromosomes are visible by interference microscopy, with H-GFP fluorescence (green/white). (B) Projections of focal sections from nuclei of the indicated genotypes showing DAPI (blue) stained chromosomes, with associated bands of H-GFP fluorescence (green/white); Hairless is located in the euchromatic regions between the densely stained sections of the chromosome. We note that an untagged wild-type copy of Hairless was also present in the experiments, because the Hairless L235D proteins are not able to rescue viability of a Hairless mutant.
(TIF)

S5 Fig Both Hairless and SMRTER contribute to repression of *reaper* and *cut* enhancers. Depletion of Hairless or SMRTER leads to de-repression of (A) *reaper11-lacZ* reporter (green, upper; white, lower) and (B) *cut1*.*3-GFP* reporter (green, upper; white, lower). Wing discs expressing the indicated RNAi’s in the e*ngrailed* domain, complementary to the regions detected by anti-Ci (purple; additional control RNAi’s were included where appropriate as summarized in methods).
(TIF)
