# Supplementary figures and images for: Role of co-repressor genomic landscapes in shaping the Notch response
Source: PLoS Genet. 2017 Nov 20;13(11):e1007096. doi: 10.1371/journal.pgen.1007096 (PMC5714389; doi:10.1371/journal.pgen.1007096)

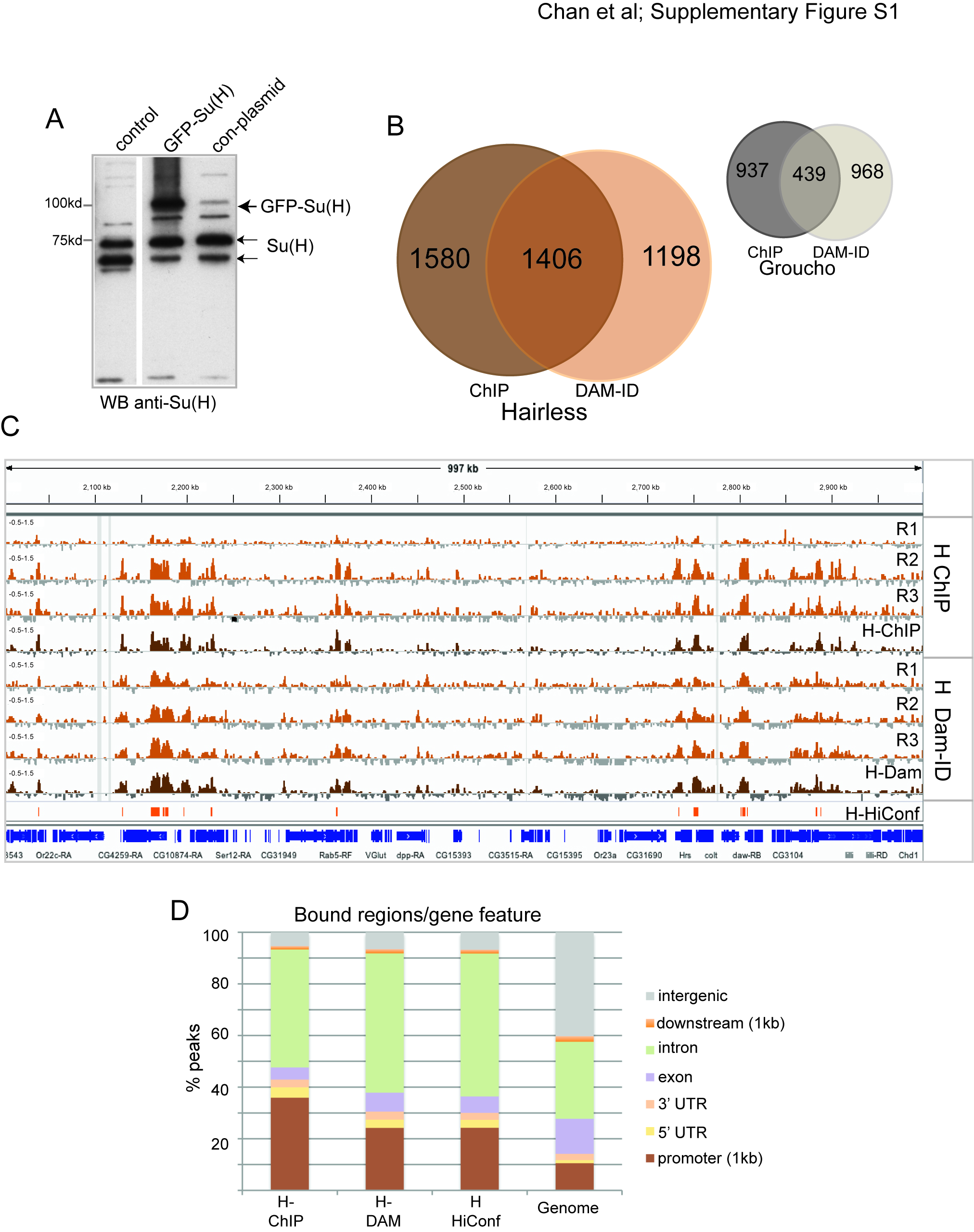

Supplement: S1 Fig — (A) Expression levels from plasmids with tagged genomic fragments are similar to endogenous levels. Western blot detecting GFP from controls (no Su(H)-GFP) and from Kc cells containing GFP-Su(H) or a control plasmid. A larger band corresponding to GFP-Su(H) (large arrow) is detected in cells containing the Su(H)-GFP plasmid, expression levels are similar to endogenous Su(H) (small arrows) present in all extracts. (B) Venn diagram illustrating overlap between regions significantly methylated by Hairless-Dam (10% FDR; pale orange) and enriched in Hairless-GFP ChIP (1% FDR; brown). The overlap is highly significant (p = 0, Fishers exact test). Inset; Overlap between similar data sets for Groucho from [6]. (C) Genomic region from 2L comparing the profiles of replicates (orange) from H-DamID (upper, R1-R3) and H-GFP ChIP (lower R1-R3) with the combined profile obtained for each dataset (brown). Fold enrichment, Log2 scale -0.50 to -1.5. Orange bars indicate regions enriched in both H-GFP ChIP and H-DamID, referred to as Hairless high confidence bound regions. Gene models are depicted in blue. (D) Percentage of peaks from the different data sets as indicated overlapping with the genomic features, coloured according to the legend. (TIF) [file pgen.1007096.s006.tif]

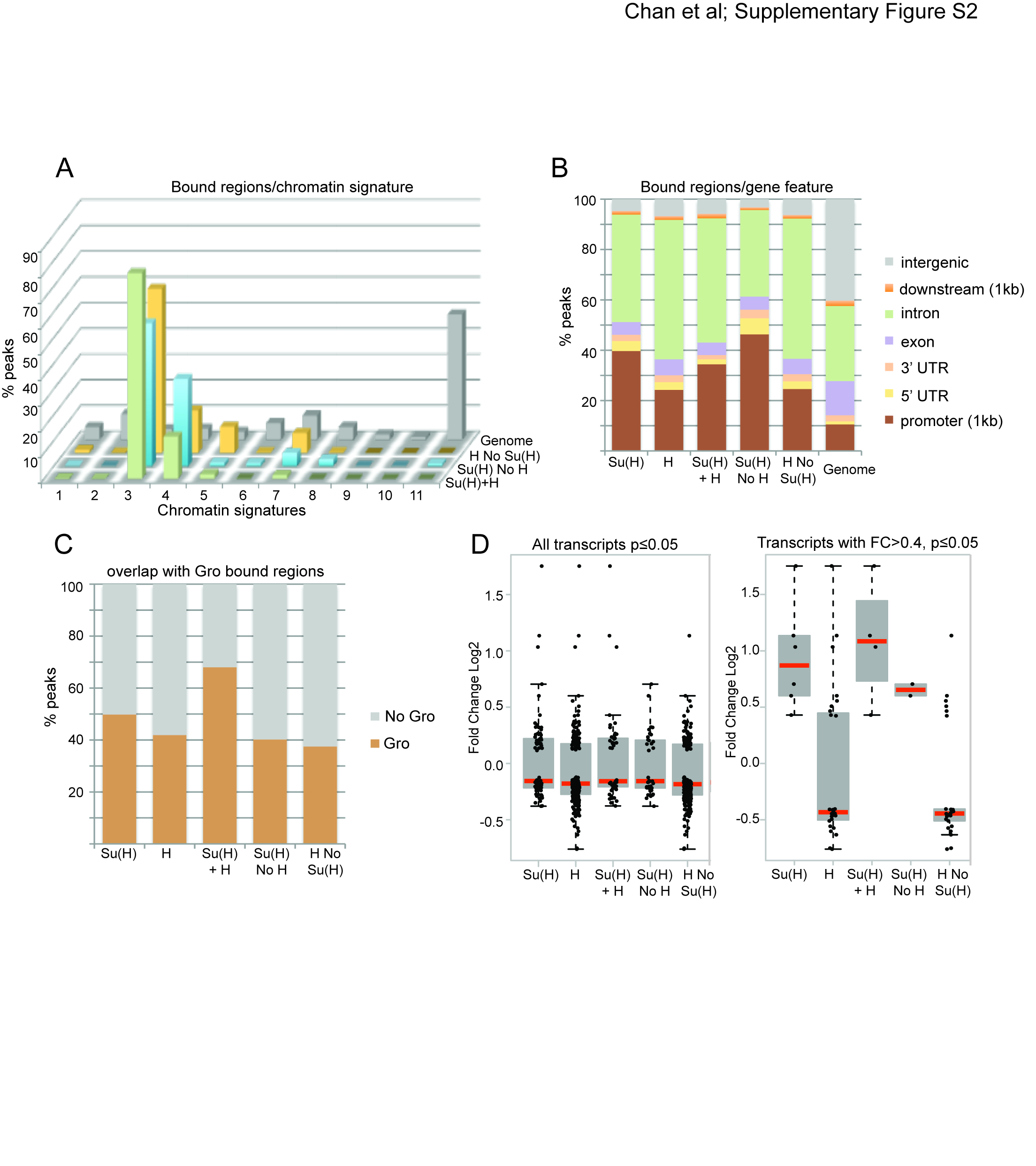

Supplement: S2 Fig — (A) Distributions of regions occupied by Su(H)+Hairless, Su(H) only or Hairless only in relation to chromatin states, all exhibit strong preference for signature 3, “enhancer” state and, to a lesser extent, signature 4 active TSS state (see methods and [34] for further details). (B) Percentage of peaks from the different data sub-sets as indicated overlapping with the genomic features, coloured according to the legend. (C) Percentage of peaks from the different indicated datasets overlapping with the regions bound by Groucho (from [6]). (D) Fold change in RNA expression levels when Hairless is depleted for genes associated with the indicated subsets of bound regions. Left graph depicts all genes with significant change in expression (p≤0.5); Right graph, only those genes with log2 fold change >0.4 (p≤0.05). (TIF) [file pgen.1007096.s007.tif]

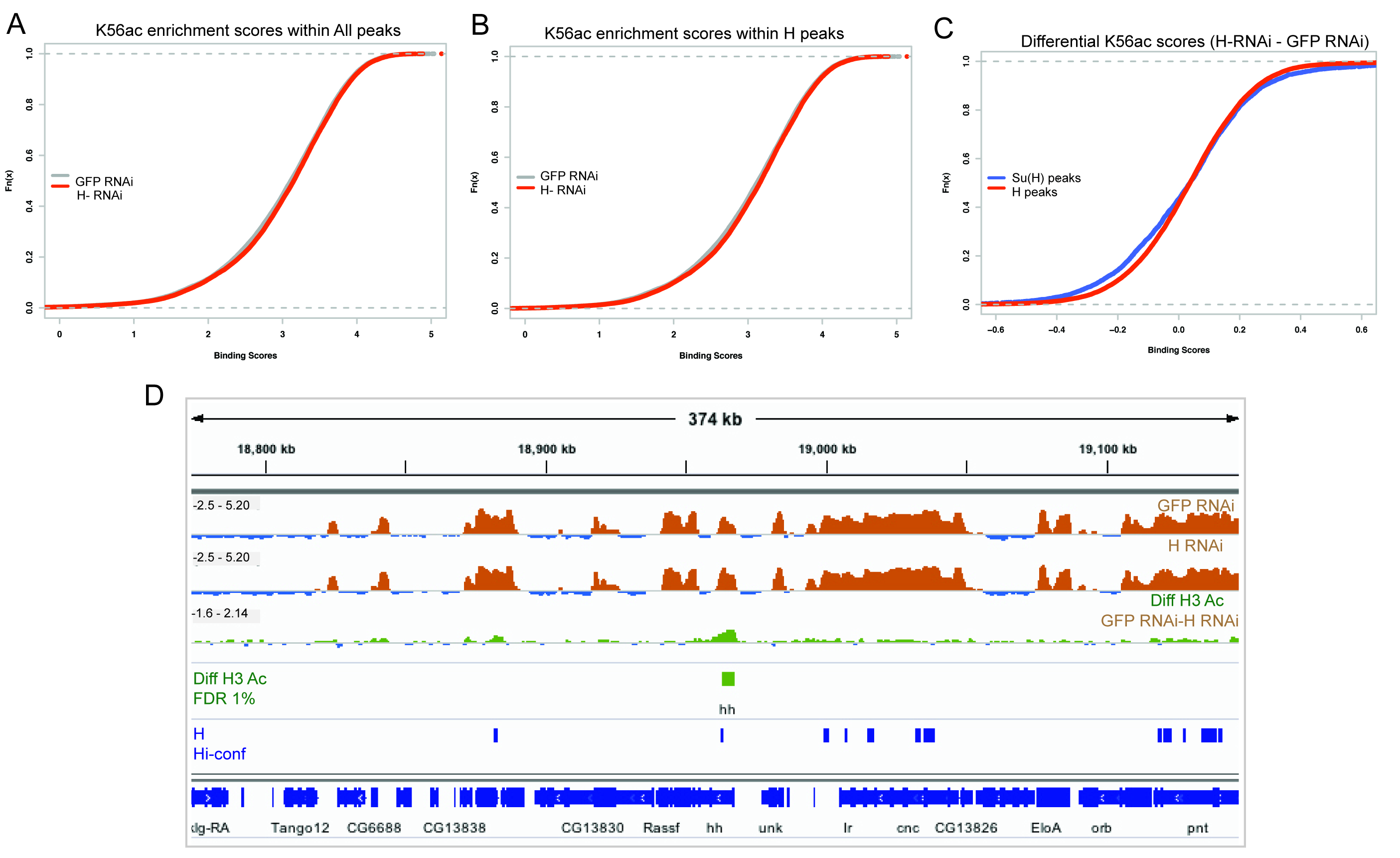

Supplement: S3 Fig — (A-B) Empirical distribution frequency plots comparing K56ac enrichment in control (GFP-RNAi) and Hairless depleted (H-RNAi) cells at all peaks and at Hairless peaks, no large-scale differences are detected. (C) Empirical distribution frequency plot comparing differential K56ac levels at H peaks (red) and Su(H) peaks, indicates that they are similar, with Su(H) peaks showing a slightly higher frequency of changes. (D) Knock-down of H results in an increase in H3 acetylation at a few H bound positions, illustrated by hh. Graphs show H3K56ac enrichment (brown) and differences in H3K56ac enrichment (green) in control and H RNAi treated Kc cells, regions of significant difference are indicated by the green box (1% FDR; see [34]). Hairless bound regions are indicated by blue boxes; not all H bound regions exhibit significant changes in H3K56ac when Hairless is depleted. (TIF) [file pgen.1007096.s008.tif]

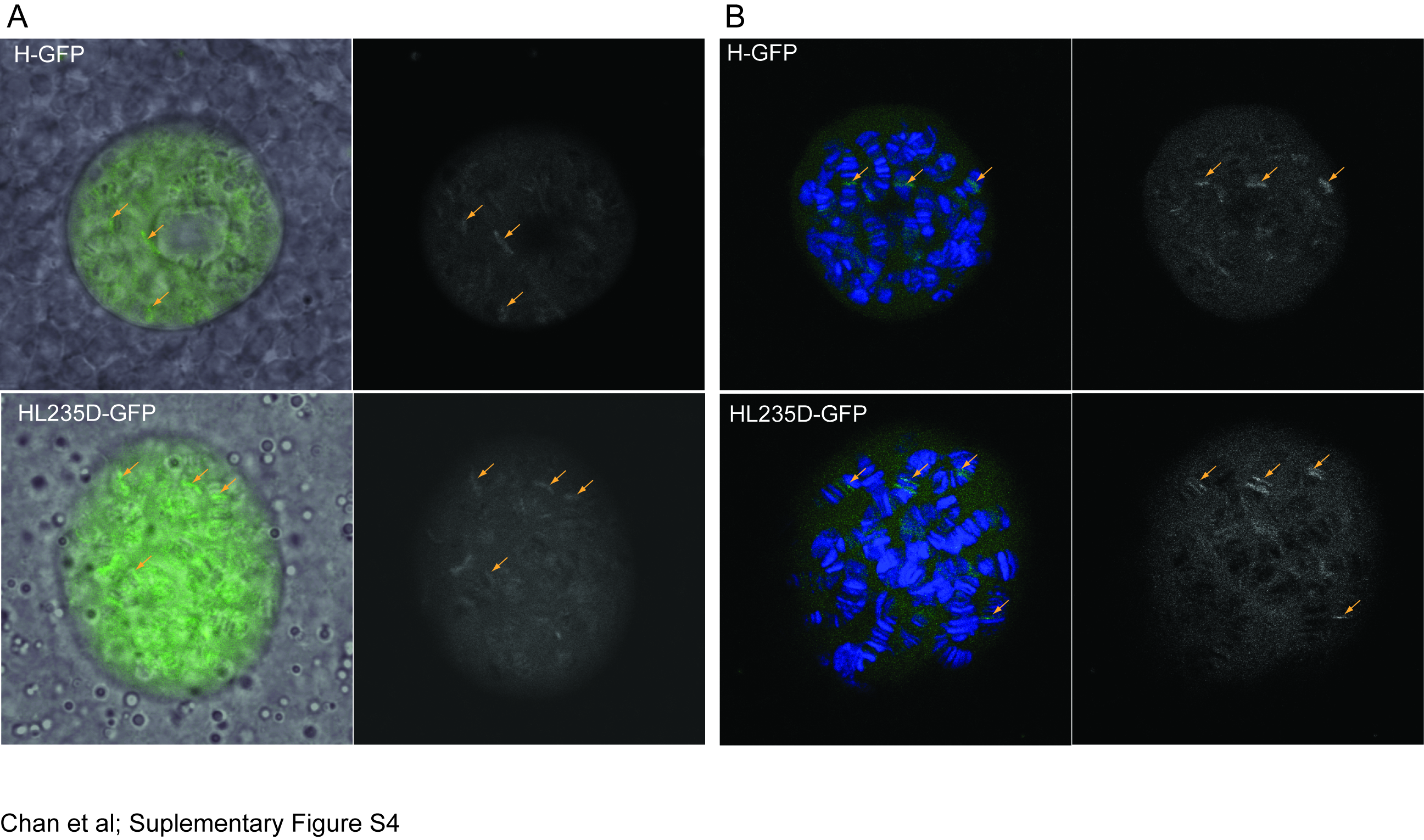

Supplement: S4 Fig — Both wildtype and Hairless L235D (a mutation that perturbs Su(H) binding, [11] are seen to form bright “bands” (e.g orange arrows) indicative of binding to the polytene nuclei, in unfixed salivary gland nuclei. (A) Projections of focal sections through nuclei of the indicated genotypes where parts of chromosomes are visible by interference microscopy, with H-GFP fluorescence (green/white). (B) Projections of focal sections from nuclei of the indicated genotypes showing DAPI (blue) stained chromosomes, with associated bands of H-GFP fluorescence (green/white); Hairless is located in the euchromatic regions between the densely stained sections of the chromosome. We note that an untagged wild-type copy of Hairless was also present in the experiments, because the Hairless L235D proteins are not able to rescue viability of a Hairless mutant. (TIF) [file pgen.1007096.s009.tif]

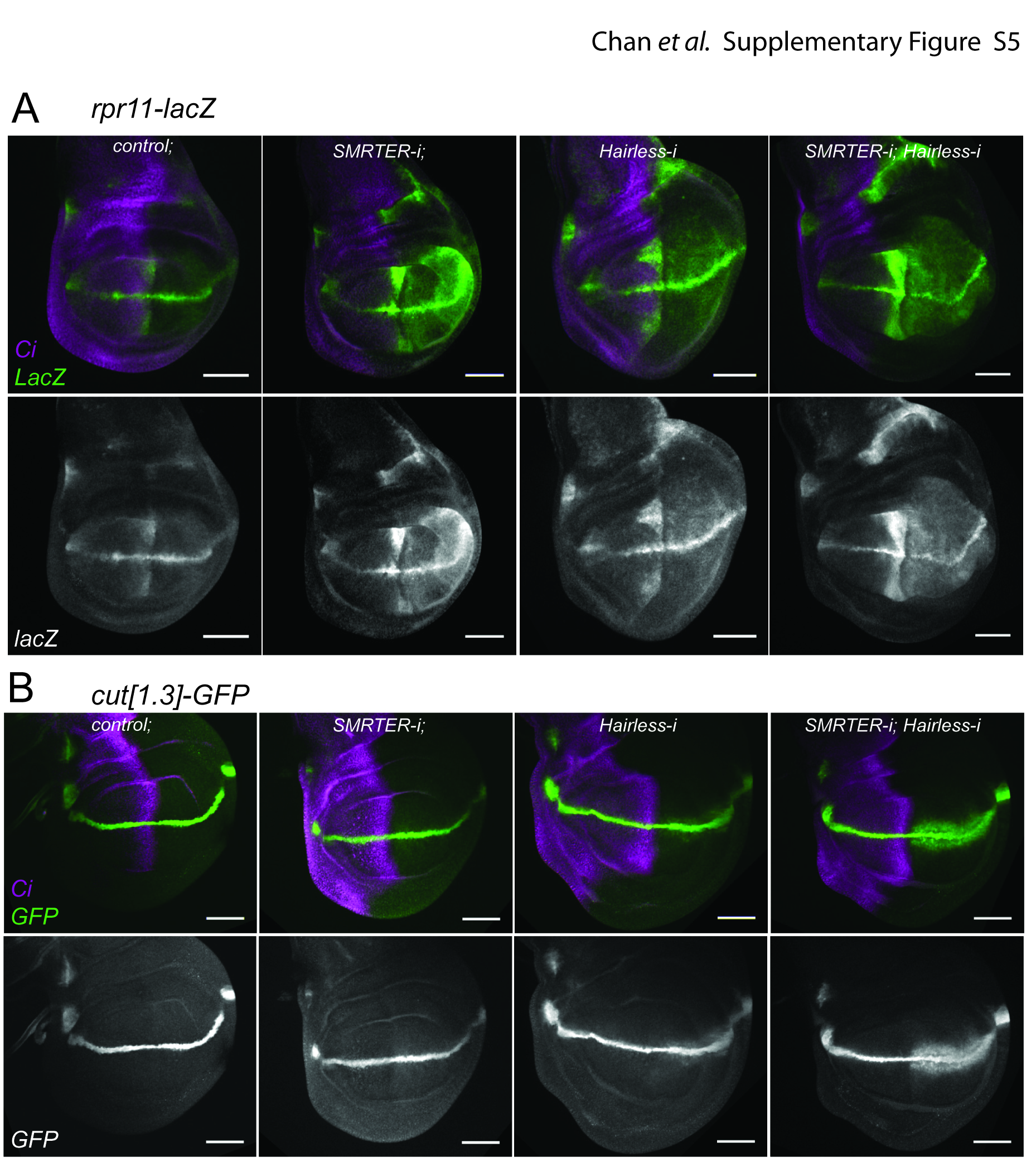

Supplement: S5 Fig — Depletion of Hairless or SMRTER leads to de-repression of (A) reaper11-lacZ reporter (green, upper; white, lower) and (B) cut1.3-GFP reporter (green, upper; white, lower). Wing discs expressing the indicated RNAi’s in the engrailed domain, complementary to the regions detected by anti-Ci (purple; additional control RNAi’s were included where appropriate as summarized in methods). (TIF) [file pgen.1007096.s010.tif]
